# Psychiatrization of Society: A Conceptual Framework and Call for Transdisciplinary Research

**DOI:** 10.3389/fpsyt.2021.645556

**Published:** 2021-06-04

**Authors:** Timo Beeker, China Mills, Dinesh Bhugra, Sanne te Meerman, Samuel Thoma, Martin Heinze, Sebastian von Peter

**Affiliations:** ^1^Department of Psychiatry and Psychotherapy, Brandenburg Medical School, Immanuel Klinik Rüdersdorf, Rüdersdorf, Germany; ^2^School of Health Sciences, City, University of London, London, United Kingdom; ^3^King's College London, Institute of Psychiatry, Psychology and Neuroscience, London, United Kingdom; ^4^School of Education, Hanze University of Applied Sciences, Groningen, Netherlands

**Keywords:** psychiatrization, transdisciplinary research, psychiatric epidemiology, medicalization, overdiagnosis, health system research, medical sociology, mental health

## Abstract

**Purpose:** Worldwide, there have been consistently high or even rising incidences of diagnosed mental disorders and increasing mental healthcare service utilization over the last decades, causing a growing burden for healthcare systems and societies. While more individuals than ever are being diagnosed and treated as mentally ill, psychiatric knowledge, and practices affect the lives of a rising number of people, gain importance in society as a whole and shape more and more areas of life. This process can be described as the progressing psychiatrization of society.

**Methods:** This article is a conceptual paper, focusing on theoretical considerations and theory development. As a starting point for further research, we suggest a basic model of psychiatrization, taking into account its main sub-processes as well as its major top-down and bottom-up drivers.

**Results:** Psychiatrization is highly complex, diverse, and global. It involves various protagonists and its effects are potentially harmful to individuals, to societies and to public healthcare. To better understand, prevent or manage its negative aspects, there is a need for transdisciplinary research, that empirically assesses causes, mechanisms, and effects of psychiatrization.

**Conclusion:** Although psychiatrization has highly ambivalent effects, its relevance mainly derives from its risks: While individuals with minor disturbances of well-being might be subjected to overdiagnosis and overtreatment, psychiatrization could also result in undermining mental healthcare provision for the most severely ill by promoting the adaption of services to the needs and desires of the rather mild cases. On a societal level, psychiatrization might boost medical interventions which incite individual coping with social problems, instead of encouraging long-term political solutions.

## Introduction: Scope of the Problem

According to the World Health Organization, nearly 10% of the world's population is affected by common mental disorders at any given time (1). Depression and anxiety disorders alone are estimated to cost the global economy more than one trillion dollars each year (2), while the burden on health-care systems and societies is allegedly still underestimated (3, 4) and projected to grow constantly (5). However, epidemiological field studies mostly suggest either consistently high prevalences since the introduction of DSM-III in the year 1980 or show rather small increases (6). In the USA, nearly a full half of the population is claimed to meet the criteria for a DSM-IV disorder over the course of their lives (7, 8), confirming previous data using DSM-III-R as diagnostic manual (9). A meta-analysis of mental health surveys across 63 countries identified an average 12-month prevalence of 17.6% for common mental disorders (10). At the same time, epidemiological research on child and adolescent mental health indicates that approximately one in five children and adolescents worldwide are affected by mental health problems (11, 12).

Despite epidemiological research pointing to high, but relatively stable incidences and prevalences of mental disorders, there is clear evidence that more and more people are using in- or outpatient mental health services, regularly resulting in the prescription of psychotropic medication. For instance, antidepressant drug consumption more than doubled between 2000 and 2015 in many OECD- countries (13). In Germany, there have been constant increases of psychiatric hospital beds and in-patient case-numbers from 2007 to 2016 (14). Also, disability pensions due to mental disorders have increased in many countries (15–17). In the USA, the number of outpatient mental health service users increased by nearly one-fifth within one decade, while an estimated 1 in 6 US-adults are on psychiatric drugs at some point per year (18, 19) Among American college students, the rates of past-year psychiatric or psychotherapeutic treatment nearly doubled from 2007 to 2017 (from 19 to 34%) (20). Epidemiological field studies concerning mental disorders in non-Western countries are rare and prevalence rates often rely on estimates (10, 21–23). Nevertheless, Western psychiatric concepts and mental healthcare are expanding to the Global South, which is supported by international organizations like the WHO and World Bank, advocating for “scaling up” access to mental health services (24).

Explanations for the consistently high prevalences, increase in diagnoses and rising mental healthcare service use are diverse. It has been recurrently argued that improved recognition and advancing destigmatization of mental disorders might be uncovering its real prevalence for the first time (25–27). Also, contemporary working and living conditions (28–32), conflict, poverty and inequality (33), inflated epidemiologic data (34, 35), and overdiagnosis (36, 37) are speculated to be contributing to what seems to be a significant increase in psychiatric morbidity. Although all of these factors appear to be relevant, the question remains whether there is a more general, higher-order process behind these developments, both connecting and explaining them. In this paper, this process is identified as a progressing psychiatrization of society.

This article aims to be a theoretical contribution to advance further studies. Its main purpose is the systematic development of a model of psychiatrization, which can serve as starting point for both empirical and conceptual research.

### Methodology (Purpose)

There is a rich canon of literature in the social sciences, medical anthropology, and critical psychology which deals with various aspects of psychiatrization, but mostly using different terminology and against different theoretical backgrounds. Also, this literature usually targets a micro-level [e.g., ethnographic case studies on the effects of psychiatric diagnosis (38–40)] or is unspecific to the field of psychiatry (e.g., research on medicalization, pharmaceuticalization, see below). Yet, as discussed in the introduction, there is empirical research from the medical field that points at various developments within psychiatry (e.g., research on drug safety, prescription rates, overdiagnosis, and overtreatment), based on numerous sources and levels of data from different domains and disciplines. Both fields of discourse are rarely brought into productive contact with each other. This may result in conceptual research which tends to neglect empirical data of the criticized psychiatric discourse itself and then again in medical research which does not specifically aim at a theory-based interpretation of its own findings in the light of larger social, political, and cultural developments.

Methodologically, this article is a conceptual paper which focusses on theoretical considerations and theory development. Its intention is neither to prove empirically that psychiatrization exists nor to speculate in whichever ontological sense this could be true. In line with Grant & Booth's typology of reviews, it can be considered as “critical review” that “goes beyond mere description” to produce “a hypothesis or a model, not an answer,” and which can serve as a “launch-pad” for further conceptual, but also empirical research [(41), p. 93]. Drawing on a diverse literature base on various aspects of psychiatrization, this article aims at mediating between the plurality of disciplines, concepts, and available data. It intends to contribute to a synthesis of the discourses within medical and social sciences, which are not only heterogeneous but stand in a tradition of being perceived as incommensurable.

Given that psychiatrization is a highly diverse, ambiguous and in itself transdisciplinary research object with fuzzy edges, a systematic literature review covering the multitude of disciplines involved was not feasible. Instead, literature was selected with regard to content and by focusing on the most influential authors and most quoted theoretical contributions surrounding psychiatrization over approximately the past 25 years. Drawing on this rich corpus of literature, an overarching, yet preliminary, model is proposed, which integrates the main actors, drivers and sub-processes of the field into a larger framework that eventually aims at setting the stage for further transdisciplinary research.

Given that the main body of research literature focusses on the Global North, the emphasis of this article will necessarily lie on how psychiatrization manifests in industrialized countries where established psychiatric services already exist. However, despite the limitations of our approach, psychiatrization in low and middle income countries will remain an equally important topic for any kind of further research.

## Conceptual Analysis

### Related Concepts

The term “psychiatrization” is first mentioned in psychiatric literature in the year 1983 by Dušan Kecmanovic, who briefly discusses psychiatric labeling of social phenomena or of deviance from existing norms (42). Within the last years, a range of popular criticism about certain aspects of psychiatry's expansion has been mostly interested in the soaring use of psychotropic medication or the inflation of diagnostic categories in the context of DSM-5, which appeared in 2013 (36, 43–46). However, more ambitious empirical and theoretical scholarship with an explicit focus on psychiatrization as higher-order process, aiming at an overarching theory or presenting a comprehensive model is rare.

For instance, the sociologist Nikolas Rose examines reasons for and interpretations of the inflation of some psychiatric diagnoses and related treatments, but without using the term psychiatrization or attempting to systemize his findings under a different term. Instead, he concludes by advocating a more complex approach to understand the growth of these diagnoses in the broader context of Western societies and their cultural developments (47). The rich work of philosopher of science Ian Hacking puts an emphasis on how psychiatric classification interacts with society, but without focusing on the expansion of psychiatry as a whole, e.g., by considering quantitative data, or developing a more unified model. However, Hacking compellingly shows how psychiatric taxonomy can deeply change the identity of the targeted persons, who, in return, react to the provided description by various degrees of embracement or resistance, which then creatively re-shapes the classifications (“looping effect”) (48, 49). Hacking also claims that classification has the power of literally “bringing into existence” the classified objects, thus “making up people” and creating “ecological niches” for new ways of existence as a certain kind of person (50, 51).

Furthermore, there is some ethnographic research detailing a few of psychiatrization's mechanisms at work in specific countries and groups worldwide (38–40, 52–54). Theoretical and experiential accounts of psychiatrization are also evident in psychiatric user and survivor scholarship, the burgeoning area of Mad Studies (55–57), and, of course, the classic anti-psychiatric literature of the 1960s and 70s (58–61). Recently, there have been several campaigns and publications addressing medical overdiagnosis and disease-mongering in general but lacking a special focus on psychiatry (62–66). Conceptually, psychiatrization unfolds as a co-production of various psy-disciplines (psychology, psychotherapy, psychoanalysis) from which mostly synergistic processes of dispersion of psy-knowledge, concepts, and vocabulary are derived (67). It shares many features with various current or preceding concepts and theories that are grounded in a plethora of disciplines:

(1) The conceptual framework of medicalization has been mainly coined by social scientists, among them Irving Zola, Peter Conrad, and Ivan Illich (68–74). Medicalization is understood as the process of defining and treating problems as medical that formerly had been perceived as non-medical, and thus expanding medical jurisdiction into new realms.

(2) Building on these ideas, the concept of biomedicalization (75) describes an intensification of medicalization driven by technological progress in the bio- and life-sciences, whose main vector of expansion is the conversion of health into a commodity and normality into something which has to be maintained or actively produced.

(3) A third line of argument uses the term pharmaceuticalization to point to a growing consumption of prescription- and lifestyle-drugs in many fields of medicine (76–78). More specific to psychiatry, medical anthropologist Janis Jenkins (79) explores how the cultural constitution of the self is influenced by widespread use of psychopharmaceuticals, while Nikolas Rose (80) has coined the expression of “neurochemical selves” for individuals who experience their own emotions as epiphenomena of their brain chemistry.

(4) A fourth theoretical tradition builds on the notion of psychologization (81, 82) or therapeutization (83), seeing psychology as a discipline that shares or better reproduces many of psychiatry's most fundamental assumptions. Yet, unlike psychiatry, psychology does not necessarily make claims about the biological base of mental illness or human behavior in general. Instead it supports the psychiatric epistemology by centering around individualist categories (e.g., individual capacities or deficiencies), and thereby tending to overlook or neglect political and social contexts. In a slightly different sense, psychologization is also used to refer to society's growing interest in individual emotions and psychological mechanisms in general over the last decades, preparing the breeding ground for what has been called a “therapy culture” (84). In a similar vein, it has been argued that many psychological concepts relating to harmful events and negative human experience have undergone semantic shifts within the last years in a way that they now include a broader range of phenomena or quantitatively less extreme examples of already known phenomena. This “concept creep” is hypothesized to mirror society's growing sensitivity toward harm and suffering, but on the other hand, criticized for contributing to further psychologizing and pathologizing normal experiences (85).

### Psychiatrization: A Working Definition

**Figure 1:**
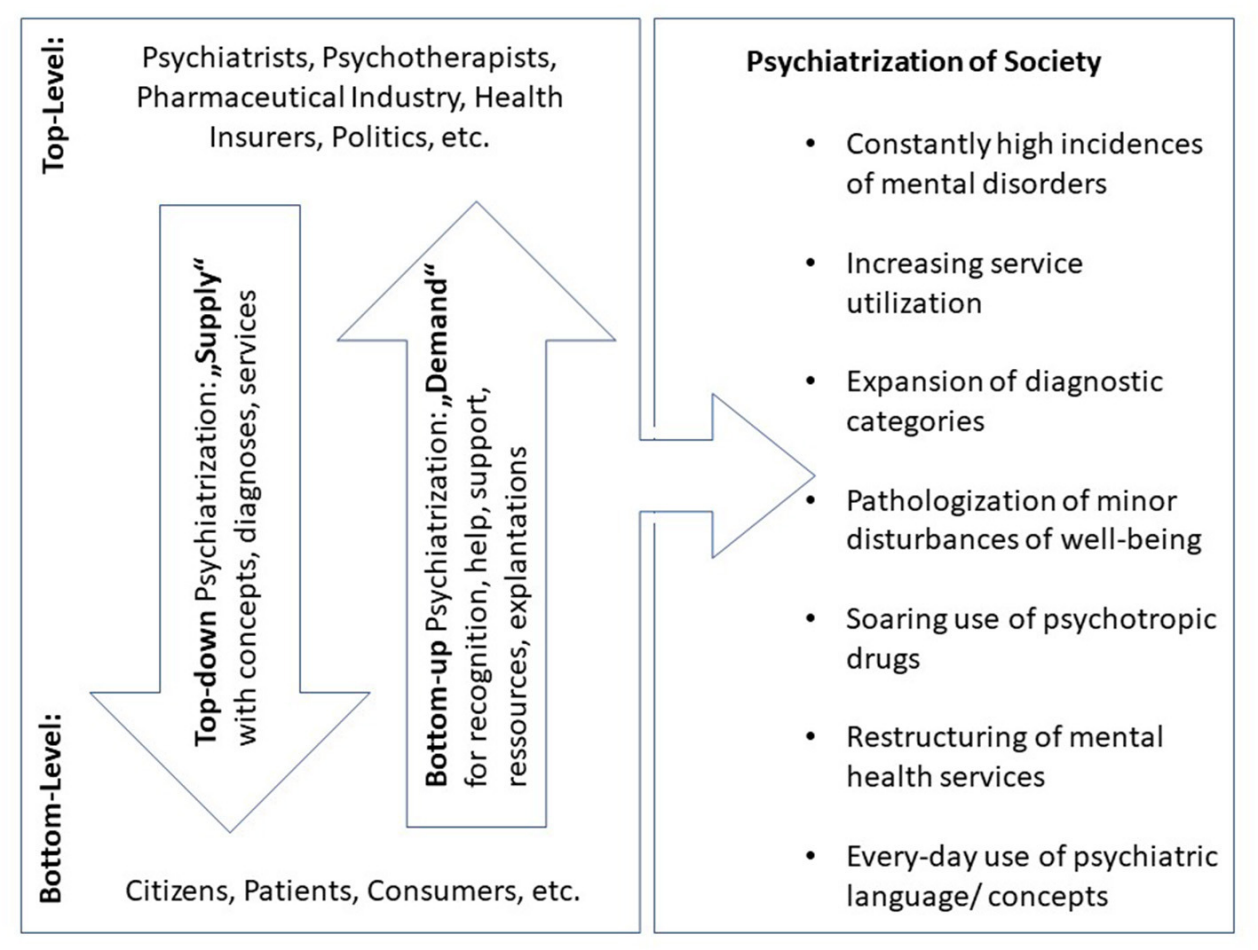
Top-down and bottom-up psychiatrization (original figure). Main protagonists and vectors of psychiatrization consisting of heterogeneous sub-processes, of which the most important are listed on the right side of the figure.

Psychiatrization is notoriously hard to define, as psychiatry itself is diverse, comprising rivaling branches with very different views on what causes and defines mental disorders and how to treat them. Also, the boundaries between psychiatry and neighboring disciplines like clinical psychology are often fuzzy and difficult to determine. Synthesizing the aforementioned approaches and concepts, we suggest to define psychiatrization as a *complex process of interaction between individuals, society, and psychiatry* through which psychiatric institutions, knowledge, and practices affect an increasing number of people, shape more and more areas of life, and further psychiatry's importance in society as a whole. Psychiatrization is an ongoing process which is not monolithic. Like other complex social developments, such as individualization or modernization, it is in itself extremely heterogeneous and appears in multiple, steadily transforming sub-processes (86) (see Figure 1). It can include both material (e.g., growth of psychiatric infrastructures) as well as ideological aspects (e.g., defining a certain condition as disorder) and is rooted in numerous fields and disciplines (e.g., psychology, psychotherapy, etc.). As a whole, psychiatrization reciprocally both causes and reflects the seemingly high incidences of psychiatric disorders and growing mental healthcare utilization.

Although there is some criticism that psychiatric epidemiology might be over-inclusive and biased (87–89), high prevalences and incidences of mental disorders, and even more so, strong evidence for growing service-use based on psychiatric diagnosis indicate that an increasing number of people currently *are* or *are likely to* be affected by psychiatry either directly or indirectly. This demonstrates the core feature of psychiatrization: its strong drive toward quantitative expansion (47), which comes into being, for example, through changes in diagnostic practices [e.g., through diagnostic inflation (36, 73, 90)], the growth of the psychiatric healthcare system in many countries or the soaring use of psychotropic drugs worldwide (13, 19, 91). Changes at the institutional or scientific level often correspond with more subtle transformations, such as the infusion of psychiatric terminology into everyday language (e.g., trauma, paranoid) or the interpretation of life events and personal experiences through the lens of psychiatric concepts (e.g., burn-out, depression). Thus, psychiatrization also transforms the life worlds of people without any personal connection to psychiatry.

However, the general psychiatrization of society might also be contrasted with rare examples of de-psychiatrization, which demonstrate that psychiatrization is not a deterministic one-way road. Instead, it is actively negotiated and can sometimes even be openly resisted by professionals as well as by laypeople. The most prominent case of de-psychiatrization might be the de-pathologization of homosexuality and its removal from DSM-II in 1973, showcasing that changing attitudes in society can also result in the rejection of psychiatric labels and normalize behavior, which was previously deemed “sick” or “ill” (92). Also, competing psy-disciplines might sometimes, at least partially, challenge psychiatrization despite sharing some underlying logic, for example, when therapists oppose the pharmaceutical treatment of disorders thought to respond better to psychotherapy.

### Approaching a Comprehensive Model

According to the literature mentioned above and the broad variety of factors for psychiatric expansion that it displays, it is important to keep in mind that the various manifestations of psychiatrization are not under central control or a common endeavor of certain key-players. Their power derives much more from public and scientific discourse or economic rationality than from deliberate actions of specific individuals. This also implies that explanations that mainly focus on the collaboration between psychiatry and the pharmaceutical industry run the risk of scientifically falling short (36, 44). However, in most cases, psychiatrization unfolds in multiple interactions with vectors going *top-down* as well as *bottom-up*. This dynamic can also implicate looping-effects in the very sense of Ian Hacking's theory (48, 49).

As a heuristic approximation, relevant protagonists can be classified into agents on the top-level or on the bottom-level. Top-level agents are defined as being either mental healthcare professionals or in other ways professionally tied to the healthcare system, while the agents on the bottom level are “laypeople” from a medical point of view, who do not have a professional connection to (mental) healthcare. A comprehensive model of psychiatrization and any further analysis needs to incorporate these two main levels and the vectors of interaction in between (see, Figure 1). This structure may also serve as a scheme to help characterize single sub-processes of psychiatrization (93).

#### Top-Down Psychiatrization

Typical protagonists of top-down psychiatrization would be psychiatrists, psychotherapists, clinical, and non-clinical scientists with connections to psychiatry, politicians with an impact on healthcare on a structural level, health insurers, pension funds, the pharmaceutical industry, and medical engineering companies. Examples for top-down-initiated psychiatrization processes can be large scale restructuring of mental health services, lawmaking, publication of new treatment and diagnostic guidelines, the introduction of new diagnoses into ICD and DSM, diagnosis- or treatment-related financial incentives by insurers, the development of new and the approval of existing psychotropic drugs for certain conditions, compulsory mental health screening in schools and workplaces, and requirements for diagnoses to access educational support.

In the texts that form the foundation for this conceptual mapping, various examples for analysis focusing on top-down-mechanisms are provided. For instance, Whitaker and Cosgrove detail how top-down psychiatrization in the USA evolved in close cooperation between the American Psychiatric Association, the pharmaceutical industry and academic psychiatry, resulting in the systematic popularization of overestimated benefits from SSRI-treatment and in official treatment guidelines comprising recommendations which contradict solid scientific evidence (44).
Conrad (72) describes the re-shaping of the DSM-III diagnosis “social phobia” into “social anxiety disorder” (SAD) in DSM-IV, which has been criticized elsewhere as “the medicalization of shyness” (94). Small changes in wording expanded the reach of this previously rather rare diagnosis considerably. This was embraced by the pharmaceutical company SmithKline Beecham's as an opportunity to sell the SSRI-antidepressant Paxil, despite the market for depression having already reached saturation. Conrad highlights the crucial importance of *lawmaking*, as the loosening of legal requirements for direct-to-consumer (“DTC”) pharmaceutical advertising in the United States set the stage for a new marketing strategy with emphasis on television commercials. These turned out to play a key role in creating the “anxiety-market” by raising public awareness for SAD as a widespread and highly debilitating condition and, after Paxil's FDA-approval for SAD in 1999, by promoting Paxil as the adequate remedy. In this case of top-down psychiatrization, changes to the DSM and federal lawmaking contributed heavily to the creation of a new epidemic of SAD with estimated point prevalences of up to 13,3% in the US-population on the one hand (72), and Paxil becoming one of the world's best-selling drugs of all time on the other hand (71).
A more recent example of top-down psychiatrization, which has been discussed extensively in both scientific and popular literature, is the suspension of the so called “bereavement-exclusion” from DSM-IV to DSM-5 (95–97). This alteration, which was performed in a top-down-way by the DSM-5 Task Force, is criticized for further inflating the psychiatric category of depression, thus blurring the line between mental illness and ordinary grief while making more individuals eligible for psychiatric treatment.
In the Global South, top-down psychiatrization may occur through turning culturally accepted ways of expressing distress into psychiatric conditions, e.g., through integration as culture-bound syndromes into DSM and through mental health legislation, such as the categorization of specific psychotropic drugs as “essential medicines” by the WHO or its encouragement of the use of the mhGAP-Intervention Guide as diagnostic and treatment algorithm in primary care (24, 98, 99). Also, the pharmaceutical industry's initiatives to open up new markets for psychotropic medication in non-Western countries can often be seen as mainly top-down driven cases of psychiatrization (100). Exporting specific medication may also entail the export of the very Western concepts of mental disorders which are the underlying rationales for its use, as has been discussed e.g., for the marketing of SSRI-antidepressants in Japan (101, 102).

#### Bottom-Up Psychiatrization

Most criticism about psychiatry's expansion highlights agents on the top-level and top-down processes. However, it seems to be a main characteristic of psychiatrization in modern and postmodern societies that it is advanced to a significant degree by laypeople without professional ties to psychiatry or the health-care system in general (see, Figure 1). This argument is in line with Michel Foucault's claim that psychiatrization might be “requested, rather than imposed” [(103), p. 296] and “does not come from above, or not only from above” [p. 295]. As opposed to top-down psychiatrization, where the supply of certain options (treatments, diagnosis, etc.) precedes and prompts the demand on the bottom-level, the concept of *bottom-up psychiatrization* underlines that the needs and desires of patients, proto-patients, and consumers can also induce changes on the top-level. This typically results in widening the range, changing the kind or facilitating the accessibility of the available options. Hence, the psychiatric permeation of individual life and collective spheres in capitalist Western societies is to a large part *demand-* and *consumer-driven*, which resonates with widespread claims about the gradual transformation of patients into consumers in medical sociology (70, 104, 105) and the commodification of individual health (75, 106).

Typical drivers of bottom-up psychiatrization might be people searching for recognition of subjective suffering or difference through clinical diagnosis (47), people with mild or unspecific “symptoms” using professional healthcare services without clear indication (107, 108), or the demand of parents or other caregivers for diagnoses and treatment of perceived learning and behavioral disorders (109, 110). Individual interests might also be organized in and articulated by advocacy groups trying to raise awareness for certain diseases and stimulate political action in favor of people with specific diagnoses (110, 111).
In the aforementioned literature and related publications, several examples for bottom-up psychiatrization can be found, although the traditional view of psychiatric expansion lays more emphasis on top-down processes. Conrad (71) and Scott (112) analyze the inclusion of Posttraumatic Stress Disorder (PTSD) into the DSM-III as a joined endeavor of returning Vietnam war veterans and some anti-war psychoanalysts and psychiatrists. The objectification of PTSD as psychiatric disorder hence was driven to a substantial degree by political motivations and private, not least financial interests of laypeople, namely ex-soldiers, whose psychological distress due to deeply disturbing war experiences had not been officially recognized as disorder before (113).
Conrad and Potter (48) describe how Attention Deficit Hyperactivity Disorder (ADHD) evolved from a condition which used to be limited to childhood into a lifespan disorder. This transformation was triggered by a wave of books and articles in lay media popularizing the idea that ADHD could persist beyond childhood and might account for many problems in adult life such as relationship issues or disorganization at the workplace. The ADHD support and advocacy group CHADD (“Children and Adults with Attention-Deficit/Hyperactivity Disorder”) played a prominent role in the further promotion of the idea that ADHD should be seen as neurobiologically caused and consequently as a lifespan-disorder. Within this context, many adults who claimed to recognize themselves in ADHD-symptomatology were seeking official confirmation of their self-diagnosis from GPs and psychiatrists, often also asking to be treated with medication. In this case, bottom-up psychiatrization was mainly driven by ordinary individuals' demand for explanations, official recognition and medical treatment of their life-problems or suffering as psychiatric disorder, relating to what Nikolas Rose has described as the readiness for “the psychiatric reshaping of discontents” [48, p. 479]. It ultimately led to the inclusion of adult ADHD into DSM-IV and the FDA-approval of psychostimulants and other medication for its treatment, which from then on were routinely prescribed by physicians.
Similar constellations, in which primarily consumers and/or patients campaign for the official recognition of particular disease entities, can be found for many psychosomatic symptom clusters such as chronic fatigue or fibromyalgia syndrome (114, 115). However, because laypeople always need to mobilize agents of the top-level to achieve effective changes (e.g., of the DSM), in all of these examples medical expertise has to be incorporated at some levels. This expertise may consist of scholars with research interest in particular conditions, or in clinicians who also identify as activists for a certain kind of suffering and become “moral entrepreneurs” [48, p. 476]. Top-level agents may thus even actively encourage bottom-up psychiatrization (93). Also, as in the case of CHADD, financial support from the pharmaceutical industry might help to maximize reach and political leverage of self-advocacy (48, 116). Still, it seems justified to interpret the above cases as bottom-up psychiatrization, as the main initiative in all of them derives from ordinary people without professional ties to the healthcare-system.
In the Global South, bottom-up psychiatrization appears to happen more rarely or is at least less represented in scientific literature. Mental health advocacy, e.g., for scaling up psychiatric services or to reduce stigma, is usually led by professionals or by human rights activists, mostly originating from countries of the Global North (24). However, a key strategy of many NGOs is to train non-specialists in tasks (diagnostics, administering medication, etc.) which are usually carried out by mental health professionals. This re-distribution of professional work known as *task-sharing* explicitly aims at laypeople acting as proxies of psychiatric experts and thus could arguably be conceived as bottom-up psychiatrization (117). Still, bottom-up psychiatrization understood in the sense of demand- or desire-driven induction of changes on the top-level seems to take place rather in consumerist societies, where economies run on evoking desires and elaborate psychiatric infrastructures already exist.

## Discussion

### Relevance and Consequences

Processes of psychiatrization are increasingly relevant in the light of a fundamental reorientation of mental healthcare provision in many countries worldwide (e.g., through digitalization, further deinstitutionalization, and the scaling up of community care), which may coincide with ongoing profound political and social changes (e.g., due to economic crisis, climate change, globalization) (86, 118–121). The extent, and dynamics of psychiatrization processes largely depend on the economic situation of a region or country, the structure of its healthcare system or cultural influences. Given the magnitude of these factors, which all include a historical dimension that adds further complexity, a full assessment of context and origins remains a challenge for future research. Also, the effects of psychiatrization are diverse, highly ambivalent, and significantly influenced by the aforementioned local factors. Individuals or groups might well-benefit from aspects of psychiatrization, as the growing mental healthcare system can also increase accessibility and provision of services that are subjectively helpful and medically clearly indicated. Thus, it can be complicated for research to distinguish legitimate attempts to meet real unmet-needs from building up infrastructures which create artificial need or promote pathologization and overtreatment of mental distress, especially in areas with little specialized care for mental disorders.

However, further research about the nuances of psychiatrization is necessary. Besides significant regional differences in its causes and mechanisms, the role of mental health professionals other than psychiatrists or psychotherapists may be a crucial, widely unexplored aspect. Given the trend to the multidisciplinary treatment of mental distress in the Global North, professions such as occupational therapists, social workers, mental health nurses, or rehabilitation counselors deserve a special focus. They may be agents who play an important role in mediating between the top- and the bottom-level of psychiatrization (see, Figure 1). On the one hand, although they do not exert the power of psychiatric diagnosis themselves, they might benefit from psychiatric expansion and their professional (group) interests might be a reinforcing factor. On the other hand, their work may also contribute to preventing psychiatrization or to mitigate its effects, e.g., by avoiding hospitalization or by empowering people in mental distress to overcome crisis without consulting a psychiatrist or psychotherapist. In this context, it will also be an important research question how the growing involvement of mental health service-users as counselors or lay-therapists in psychiatric institutions relates to psychiatrization.

Advancing research on psychiatrization may be important, in the light of its obvious risks on the individual, societal and public health-level: First, on the individual level, negative consequences of psychiatrization may relate to overdiagnosis and overtreatment, e.g. medication adverse effects and harms from long term use (43, 122–128), but may also be about the impacts of labeling and of coercive treatments (129–131). Through pathologization of minor disturbances of well-being, individual variation and numerous life issues, psychiatrization can also co-produce avoidable patient careers, create dependencies on mental health services, and ultimately promote disempowering changes to subjectivity and sense of self (80, 132–134).

Second, on the societal level, psychiatrization may risk to further narrow the range of what is perceived as “normal,” encourage ineffective and short-term medical interventions, prompt individuals to cope with social problems and impede the finding of adequate long-term solutions (67, 134, 135). Such solutions would be situated rather in the realm of politics, where psychiatrization might otherwise be contributing to disguising failed policies.

Third, from a public health perspective, psychiatrization of society runs the risk of establishing widespread inverse care by increasingly neglecting the most severely and chronically ill, when mental health services are tailored to the needs of the mildly ill and borderline cases (18, 136, 137). Accordingly, the relative shortage of psychotherapists and long waiting times for out-patient services in some countries of the Global North may be a direct effect of structurally induced healthcare over-utilization by the “worried well” (138, 139).

Fourth, from a global perspective, psychiatrization could lead to excessive diagnosis and prescription of medication with little monitoring once people are medicated in countries with low and middle incomes, where psychiatrization is to a large degree exerted through task-sharing. In these countries this may also undermine local support systems and promote individualized interventions into poverty (67, 140). Worldwide, psychiatrization could contribute to challenging public health by misallocating scarce resources toward biomedical research and pharmacological treatment instead of strengthening psycho-social interventions (141, 142).

## Conclusion and Perspectives

Psychiatrization is a highly complex and diverse global process with various protagonists. Its effects are ambivalent but can be harmful in many ways to individuals, societies, and public healthcare systems. To better understand, and also to deal with negative consequences of psychiatrization, there is primarily a need for research, which might be accompanied by public debate and, ultimately, may help inform political decision-making.

On the scientific level, transdisciplinary research is necessary to empirically establish the existence of psychiatrization by assessing and, wherever possible, measuring its different causes, mechanisms and effects in relation to clearly defined areas, such as a region, a city or a nation. This kind of research should also include the different perspectives of a broad variety of professions involved in mental health care, among them, apart from psychiatrists and psychotherapists, social workers, occupational therapists, mental health nursing professionals, or rehabilitation counselors. To this end and due to the complex and multi-layered nature of the research topic, a mixed-methods approach seems most suitable (143): Quantitative methods can contribute to establishing a solid fact base about the growth of psychiatric infrastructures, local trends in prevalence, and healthcare utilization (144). Relevant data would comprise changes in treatment capacities and utilization of psychiatric hospitals and outpatient-departments, government and health insurance expenditure for mental health, trends in psychotropic drug prescription and self-reported usage, availability and utilization of psychological treatment, numbers of primary-care physician contacts for psychological problems and all kinds of available data sets about prevalence and incidences of mental disorders, e.g., as measured by national mental health surveys. Qualitative approaches such as expert interviews, in-depth group interviews or participant observation would be used to make visible the effects of psychiatrization in the everyday life of individuals, exploring subjective and collective meanings of different aspects of psychiatrization and identifying motives for engaging in psychiatrization processes or resisting them (145–147).

Both types of research will be necessary prerequisites for data-based theory development about psychiatrization, its causes, its mechanisms, and its effects on public health, individuals and society. As mentioned above, the extent and type of psychiatrization processes largely depend on the economic and political situation, culture and history of a region or country. This renders a complete assessment of context difficult to achieve. Nonetheless, a data-based theory, enriched with an in-depth description of contextual factors, seems to be a realistic goal which can also help inform public debate, stakeholders in healthcare and political decision-makers. Main overall research goals will be to better understand how changes on the level of mental healthcare provision or utilization (1) are shaped by individual action of both top- and bottom-level agents, (2) are affecting patients' and proto-patients' lives, e.g., through (over)diagnosis, changing self-definitions or inducement of patient-careers, (3) advance the dissemination of psychiatric concepts, knowledge and epistemologies in society, (4) induce or intensify the permeation of certain areas of private and public life, and (5) interact with or are determined by larger economic, social, and cultural developments.

As psychiatrization is transdisciplinary as a research-object, expertise from various fields other than psychiatry are required, such as health services research, epidemiology, and public health. To mediate between the discourses of the various sciences and disciplinary traditions, researchers with a background in ethnology, medical anthropology, sociology, and philosophy etc. should also be involved from the beginning. It will be equally important for all research to build up collaborative projects between professionals and service users that value user, survivor and Mad Studies knowledge, whose common point of reference are negative experiences with ideology and practice of clinical psychiatry and its impacts on personal well-being and biography. Thus, there is an intrinsically critical view on psychiatrization contained in the experiential knowledge of service users and the epistemologies derived from it (148).

Such transdisciplinary research as described could result in empirically proving that psychiatrization exists, developing valid indicators for its extent, showing hot spots and key-factors on a local scale, thickening theory and generating hypotheses and research goals for more complex, larger scale research programs. In the long run, as psychiatrization occurs globally, both local and global perspectives will be required, pointing out the many different ways that psychiatrization manifests, is embraced, appropriated, or resisted around the world.

## Author Contributions

TB, ST, and SP initiated research and were responsible for devising the article. TB developed the comprehensive model, wrote the initial draft, and coordinated the other authors' contributions. All authors contributed to literature search, interpretation of literature, helped draft the final version of the manuscript and revised the article critically for important content. All authors approve the final version to be published and agree to be accountable for all aspects of the work, its accuracy and integrity.

## Conflict of Interest

The authors declare that the research was conducted in the absence of any commercial or financial relationships that could be construed as a potential conflict of interest.

## References

[1] World Health Organization. Fact Sheet Mental Disorders. (2019). Available online at: http://www.who.int/mediacentre/factsheets/fs396/en/ (accessed May 12, 2020).

[2] World Health Organization. Out of the Shadow: Making Mental Health a Global Priority. (2016). Available online at: https://www.who.int/mental_health/advocacy/wb_background_paper.pdf?ua=1 (accessed May 19, 2021).

[3] Vigo D Thornicroft G Atun R. Estimating the true global burden of mental illness. Lancet. (2016) 3:171–8. 10.1016/S2215-0366(15)00505-2 26851330

[4] Takayanagi Y Spira AP Roth KB Gallo JJ Eaton WW Mojtabai R. Accuracy of reports of lifetime mental and physical disorders: results from the baltimore epidemiological catchment area study. JAMA Psychiatr. (2014) 71:273–80. 10.1001/jamapsychiatry.2013.3579 24402003 PMC4135054

[5] Bloom DE Cafiero ET Jané-Llopis E Abrahams-Gessel S Bloom LR Fathima S. The Global Economic Burden of Noncommunicable Diseases. Geneva: World Economic Forum (2011). Available online at: http://www3.weforum.org/docs/WEF_Harvard_HE_GlobalEconomicBurdenNonCommunicableDiseases_2011.pdf (accessed May 4, 2020).

[6] Richter D Wall A Bruen A Whittington R. Is the global prevalence rate of adult mental illness increasing? Systematic review and meta-analysis. Acta Psychiatr Scand. (2019 140:393–407. 10.1111/acps.13083 31393996

[7] Kessler RC Berglund P Demler O Jin R Merikangas KR Walters EE. Lifetime prevalence and age-of-onset distributions of DSM-IV disorders in the National Comorbidity Survey replication. Arch Gen Psychiatry. (2005) 62:593–603. 10.1001/archpsyc.62.6.593 15939837

[8] National Institute of Mental Health. Statistics mental illness. (2019). Available online at: https://www.nimh.nih.gov/health/statistics/mental-illness.shtml (accessed May 16, 2020).

[9] Kessler RC McGonagle KA Zhao S Nelson CB Hughes M Eshleman S. Lifetime and 12-month prevalence of DSM-III-R psychiatric disorders in the united states: results from the national comorbidity survey. Arch Gen Psychiatry. (1994) 51:8–19. 10.1001/archpsyc.1994.03950010008002 8279933

[10] Steel Z Marnane C Iranpour C Chey T Jackson JW Patel V. The global prevalence of common mental disorders: a systematic review and meta-analysis 1980-2013. Int J Epidemiol. (2014) 43:476–93. 10.1093/ije/dyu038 24648481 PMC3997379

[11] Kieling C Baker-Henningham H Belfer M Conti G Ertem I Omigbodun O. Child and adolescent mental health worldwide: evidence for action. Lancet. (2011) 378:1515–25. 10.1016/S0140-6736(11)60827-1 22008427

[12] Collishaw S. Annual research review: secular trends in child and adolescent mental health. J Child Psychol Psychiatr. (2015) 56:370–93. 10.1111/jcpp.12372 25496340

[13] Organisation for Economic Co-Operation and Development (2020) Statistics Health Status. Available online at: https://stats.oecd.org/index.aspx?DataSetCode=HEALTH_STAT# (accessed May 16, 2020).

[14] Thom J Bretschneider J Kraus N Handerer J Jacobi F. Versorgungsepidemiologie psychischer Störungen. Bundesgesundheitsbl. (2019) 62:128–39. 10.1007/s00103-018-2867-z 30635694

[15] Viola S Moncrieff J. Claims for sickness and disability benefits owing to mental disorders in the UK: trends from 1995 to 2014. BJPsych Open. (2016) 2:18–24. 10.1192/bjpo.bp.115.002246 27703749 PMC4995588

[16] Schofield TP Kiely KM Mykletun A Harvey SB Butterworth P. Using longitudinal survey data to estimate mental health related transitions to a disability pension. J Occup Environ Med. (2018) 60:166–72. 10.1097/JOM.0000000000001269 29280770

[17] Portal für psychische Gesundheit am Arbeitsplatz. Daten und Fakten. (2019). Available online at: https://www.psyga.info/psychische-gesundheit/daten-fakten/ (accessed 13 May, 2020).

[18] Olfson M Wang S Wall M Marcus SC Blanco C. Trends in serious psychological distress and outpatient mental health care of US adults. JAMA Psychiatr. (2019) 76:152–61. 10.1001/jamapsychiatry.2018.3550 30484838 PMC6439744

[19] Moore TJ Mattison DR. Adult utilization of psychiatric drugs and difference by sex, age and race. JAMA Intern Med. (2017) 177:274–5. 10.1001/jamainternmed.2016.7507 27942726

[20] Lipson SK Lattie EG Eisenberg D. Increased rates of mental health service utilization by U.S. College students: 10-year population-level trends (2007-2017). Psychiatr Serv. (2019) 70:60–3. 10.1176/appi.ps.201800332 30394183 PMC6408297

[21] Baxter A Scott K Vos T Whiteford H. Global prevalence of anxiety disorders: a systematic review and meta-regression. Psychol Med. (2013) 43:897–910. 10.1017/S003329171200147X 22781489

[22] Whiteford HA Degenhardt L Rehm J Baxter AJ Ferrari AJ Erskine HE. Global burden of disease attributable to mental and substance use disorders: findings from the Global Burden of Disease Study 2010. Lancet. (2013) 382:1575–86. 10.1016/S0140-6736(13)61611-6 23993280

[23] Ferrari A Somerville A Baxter A Pacella RE Patten S Vos T. Global variation in the prevalence and incidence of major depressive disorder: a systematic review of the epidemiological literature. Psychol Med. (2013) 43:471–81. 10.1017/S0033291712001511 22831756

[24] Mills C. Decolonizing global mental health: the psychiatrization of the majority world. London /New York, NY: Routledge (2014). 10.4324/9780203796757

[25] Richter D Berger K. Are mental disorders increasing? Update of a systematic review on repeated cross-sectional studies. Psychiat Prax. (2013) 40:176–82. 10.1055/s-0032-1333060 23564356

[26] Mars B Heron J Kessler D Davies NM Martin RM Thomas KH. Influences on antidepressant prescribing trends in the UK: 1995–2011. Soc Psychiatry Psychiatr Epidemiol. (2017) 52:193–200. 10.1007/s00127-016-1306-4 27885400 PMC5329088

[27] Mojtabai R. Mental illness stigma and willingness to seek mental health care in the European Union. Soc Psychiatry Psychiatr Epidemiol. (2010) 44:705–12. 10.1007/s00127-009-0109-2 19680588

[28] Dittmar H Bond R Hurst M Kasser T. The relationship between materialism and personal well-being: a meta-analysis. J Pers Soc Psychol. (2014) 107:879–924. 10.1037/a0037409 25347131

[29] Eckersley R. Is modern Western culture a health hazard? Int J Epidemiol. (2005) 35:252–8. 10.1093/ije/dyi235 16303804

[30] Rosa H. Social Acceleration. A New Theory of Modernity. New York, NY: Columbia University Press (2015).

[31] Cvetkovich A. Depression. A Public Feeling. Durham/London, Duke University Press (2012). 10.1215/9780822391852

[32] Ehrenberg A. The Weariness of the Self: Diagnosing the History of Depression in the Contemporary Age. Montreal, McGill-Queen's University Press (1998).

[33] Patel V Saxena S Lund C Thornicroft G Baingana F Bolton P. The Lancet Commission on global mental health and sustainable development. Lancet. (2018) 392:1553–98. 10.1016/S0140-6736(18)31612-X 30314863

[34] Horwitz AC Wakefield JC. The epidemic in mental illness: clinical fact or survey artefact. Contexts. (2006) 5:19–23. 10.1525/ctx.2006.5.1.19

[35] Brhlikova P Pollock AM Manners R. Global burden of disease estimates of depression-how reliable is the epidemiological evidence? J R Soc Med. (2011) 104:25–34. 10.1258/jrsm.2010.100080 21205775 PMC3014564

[36] Frances A. Saving normal: an insider's revolt against out-of-control psychiatric diagnosis, DSM-5, Big Pharma, and the medicalization of ordinary Life. New York, NY: William Morrow (2013).

[37] Moynihan R Doust J Henry D. Preventing overdiagnosis: how to stop harming the healthy. BMJ. (2012) 344:e3502. 10.1136/bmj.e3502 22645185

[38] Jain S Orr DM. Ethnographic perspectives on global mental health. Transcult Psychiatr. (2016) 53:685–95. 10.1177/1363461516679322 28317467

[39] Behrouzan O. Prozak Diaries: Psychiatry and Generational Memory in Iran. Bloomington, Stanford University Press (2016). 10.1515/9780804799591

[40] LeFrançois BA. The psychiatrization of our children, or, an autoethnographic narrative of perpetuating First Nations genocide through ‘benevolent' institutions. Decolon Indigen Educ Soc. (2013) 2:108–23. Available online at: https://jps.library.utoronto.ca/index.php/des/article/view/18687/16239 (accessed May 19, 2021).

[41] Grant MJ Booth A. A typology of reviews: an analysis of 14 review types and associated methodologies. Health Inform Libraries J. (2009) 26:91–108. 10.1111/j.1471-1842.2009.00848.x 19490148

[42] Kecmanović D. Psychiatrization: a general view. Int J Soc Psych. (1983) 29:308–12. 10.1177/002076408302900411 6642923

[43] Whitaker R. Anatomy of an Epidemic: Magic Bullets, Psychiatric Drugs, and the Astonishing Rise of Mental Illness in America. New York, NY: Broadway Books (2010). 10.3109/01612840.2012.713447 23017048

[44] Cosgrove L Whitaker R. Psychiatry Under the Influence. Institutional Corruption, Social Injury, and Prescriptions for Reform. New York, NY: Palgrave Macmillan (2015).

[45] Paris J. Overdiagnosis in Psychiatry. How Modern Psychiatry Lost Its Way While Creating a Diagnosis for Almost All of Life's Misfortunes. New York, NY: Oxford University Press (2015). 10.1093/med/9780199350643.001.0001

[46] Kirsch I. The emperor's New Drugs. Exploding the Antidepressant Myth. London, Random House (2009).

[47] Rose N. Disorders without borders? The expanding scope of psychiatric practice. BioSocieties. (2006) 1:465–84. 10.1017/S1745855206004078

[48] Hacking I. Rewriting the Soul: Multiple Personality and the Sciences of Memory. New Jersey, NJ: Princeton University Press (1995). 10.1515/9781400821686

[49] Hacking I. The Social Construction of What? Cambridge, Harvard University Press (1999).

[50] Hacking I. Mad Travelers: Reflections on the Reality of Transient Mental Illnesses. Charlottesville: University of Virginia Press (1998).

[51] Hacking I. Making Up People. In: Heller TL Sosna M Wellbery DE editors. Reconstructing Individualism. Stanford, CA: Stanford University Press (1985). p. 222–36.

[52] Lakoff A. Pharmaceutical Reason: Knowledge and Value in Global Psychiatry. Cambridge, Cambridge University Press (2006). 10.1017/CBO9780511489150

[53] Mills C. The Psychiatrization of poverty: rethinking the mental health-poverty nexus. Soc Personal Psychol Compass. (2015) 9:213–22. 10.1111/spc3.12168

[54] Cohen BM. Passive-aggressive: Māori resistance and the continuance of colonial psychiatry in Aotearoa New Zealand. Disabil Global South. (2014) 1:319–39. Available online at: https://disabilityglobalsouth.files.wordpress.com/2012/06/dgs-01-02-06.pdf (accessed May 19, 2021).

[55] Burstow B LeFrançois BA Diamond S. Psychiatry Disrupted: Theorizing Resistance and Crafting the (R)evolution. Montreal: McGill-Queen's University Press (2014).

[56] LeFrançois BA Menzies R Reaume G editor. Mad Matters: A critical reader in Canadian Mad Studies. Toronto, ON: Canadian Scholars Press Inc, (2013).

[57] Russo J Sweeney A. Searching for a Rose Garden: Challenging Psychiatry, Fostering Mad Studies. Monmouth: PCCS Books (2017).

[58] Goffman E. Asylums: Essays on the Social Situation of Mental Patients and Other Inmates. New York, NY: Anchor Books (1961).

[59] Foucault M. Madness and Civilization. A History of Insanity in the Age of Reason. New York, NY: Random House (1965).

[60] Szasz TS. The Myth of Mental Illness: Foundations of a Theory of Personal Conduct. New York, NY: Harper & Row (1974).

[61] Cooper D. Psychiatry and Anti-Psychiatry. London, Tavistock/Paladin (1967).

[62] Preventing Overdiagnosis: Winding Back the Harms of too Much Medicine. Available online at: https://www.preventingoverdiagnosis.net/ (accessed May 19, 2021).

[63] ABIM-Foundation Our Mission. Available online at: http://www.choosingwisely.org/about-us/ (accessed at: May 16, 2020).

[64] BMJ's Too Much Medicine Initiative. Available online at: http://www.bmj.com/too-much-medicine (accessed at: May 16, 2020).

[65] Moynihan R Heath I Henry D. Selling sickness: the pharmaceutical industry and disease mongering. BMJ. (2002) 324:886–91. 10.1136/bmj.324.7342.886 11950740 PMC1122833

[66] Moynihan R Cassels A. Selling sickness. New York, NY: Nation Books (2005). 10.1037/e514562004-001

[67] Klein E Mills C. Psy-expertise, therapeutic culture and the politics of the personal in development. Third World Quart. (2017) 38:1990–2008. 10.1080/01436597.2017.1319277

[68] Zola IK. Medicine as an institution of social control. Soc Rev. (1972) 4:487–504. 10.1111/j.1467-954X.1972.tb00220.x 4645802

[69] Conrad P. Medicalization and social control. Annu Rev Sociol. (1992) 18:209–32. 10.1146/annurev.so.18.080192.001233

[70] Conrad P. The shifting engines of medicalization. J Health Soc Behav. (2005) 46:3–14. 10.1177/002214650504600102 15869117

[71] Conrad P. The medicalization of society. On the Transformation of Human Conditions into Treatable Disorders. Baltimore, John Hopkins University Press (2007).

[72] Conrad P. The medicalization of mental disorder. In: Aneshensel CS editors. Handbook of the Sociology of Mental Health, 2nd Edn. Springer, Dordrecht (2013). p. 61–73 10.1007/978-94-007-4276-5_4

[73] Conrad P Potter D. From hyperactive children to ADHD adults: Observations on the expansion of medical categories. Soc Probl. (2000) 47:59–82. 10.2307/3097135

[74] Illich I. Medical Nemesis. London: Calder & Boyars (1974). 10.1016/S0140-6736(74)90361-4 4133432

[75] Clarke A Shim J Mamo L Fosket J Fishman J. Biomedicalization: technoscientific transformations of health, illness, and U.S. biomedicine. Am Soc Rev. (2003) 68:161–94. 10.2307/1519765

[76] Fox NJ Ward KJ. Pharma in the bedroom…and the kitchen….The pharmaceuticalisation of daily life. Sociol Health Illn. (2008) 30:856–68. 10.1111/j.1467-9566.2008.01114.x 18761507

[77] Abraham J. Pharmaceuticalization of society in context: theoretical, empirical and health dimensions. Sociology. (2010) 44:603–22. 10.1177/0038038510369368

[78] Healy D. Pharmageddon. Berkerley, LA: University of Virginia Press (2012). 10.1525/9780520951815

[79] Jenkins JH editor. Pharmaceutical Self: The Global Shaping of Experience in an Age of Psychopharmacology. Santa Fe: SAR Press (2011).

[80] Rose N. Neurochemical selves. Society. (2003) 6:46–59. 10.1007/BF02688204

[81] Gordo Lopez A De Vos J. Psychologism, psychologising and de-psychologisation. Ann Rev Crit Psychol. (2010) 8:3–7. Available online at: https://thediscourseunit.files.wordpress.com/2016/05/arcp8full.pdf (accessed May 19, 2021).

[82] De Vos J. From Milgram to Zimbardo: the double birth of postwar psychology/psychologization. Hist Hum Sci. (2010) 23:156–75. 10.1177/0952695110384774 21322973

[83] Sommers CH Satel S. One Nation Under Therapy: How the Helping Culture is Eroding Self-Reliance, New York, NY: St Martin's Press (2005).

[84] Furedi F. Therapy Culture: Cultivating Vulnerability in an Uncertain age. London, Routledge (2004).

[85] Haslam N. Concept creep: psychology's expanding concepts of harm and pathology. Psychol Inq. (2016) 27:1–17. 10.1080/1047840X.2016.1082418

[86] Bauman Z. Liquid Modernity. Cambridge: Polity Press (2000).

[87] Jorm AF. National surveys of mental disorders: Are they researching scientific facts or constructing useful myths? Aust N Z J Psychiatr. (2006) 40:830–4. 10.1080/j.1440-1614.2006.01901.x 16959008

[88] Wakefield JC Schmitz MF. The challenge of measurement of mental disorder in community surveys. In: Pilgrim D Rogers A Pescosolido B editors. The Sage Handbook of Mental Health and Illness. New York, NY: Sage (2011). p. 26–48. 10.4135/9781446200988.n2

[89] Wakefield JC. DSM-5, psychiatric epidemiology and the false positives problem. Epidemiol Psychiatr Sci. (2015) 24:188–96. 10.1017/S2045796015000116 25675983 PMC6998664

[90] Batstra L Frances A. DSM-V further inflates attention deficit hyperactivity disorder. J Nerv Ment Dis. (2012) 200:486–8. 10.1097/NMD.0b013e318257c4b6 22652611

[91] Pratt LA Brody DJ Gu Q. Antidepressant Use Among Persons Aged 12 and Over: United States, 2011–2014. NCHS Date Brief No. 283 (2017). Available online at: https://www.cdc.gov/nchs/data/databriefs/db283_table.pdf#4 (accessed May 16, 2020). 29155679

[92] Drescher J. Out of DSM: depathologizing homosexuality. Behav Sci. (2015) 5:565–75. 10.3390/bs5040565 26690228 PMC4695779

[93] Beeker T Witeska-Młynarczyk A te Meerman S Mills C. Psychiatrization of, with and by children: drawing a complex picture. Glob Stud Child. (2020) 10:12–25. 10.1177/2043610619890074

[94] Scott S. The medicalisation of shyness: from social misfits to social fitness. Sociol Health Illn. (2006) 28:133–53. 10.1111/j.1467-9566.2006.00485.x 16509950

[95] Wakefield JC. The DSM-5 debate over the bereavement exclusion: psychiatric diagnosis and the future of empirically supported treatment. Clin Psychol Rev. (2013) 33:825–45. 10.1016/j.cpr.2013.03.007 23706392

[96] Bandini J. The medicalization of bereavement: (Ab)normal Grief in the DSM-5. Death Stud. (2015) 39:347–52. 10.1080/07481187.2014.951498 25906168

[97] Horwitz AV. The DSM-5 and the continuing transformation of normal sadness into depressive disorder. Emot Rev. (2015) 7:209–15. 10.1177/1754073915575401

[98] World Health Organization. mhGAP Intervention Guide for Mental, Neurological and Substance Use Disorders in Non-Specialized Health Settings. (2010). Available online at: https://www.who.int/mental_health/publications/mhGAP_intervention_guide/en/ (accessed at: May 8, 2020). 23741783

[99] Dua T Sharma A Patel A Hanna F Chowdhary N Saxena S. Integrated care for mental, neurological and substance use disorders in non-specialized health settings: rising to the challenge. World Psychiatr. (2017) 16:216–7. 10.1002/wps.20430 28498574 PMC5428190

[100] Watters E. Crazy Like Us: The Globalization of the American Psyche. New York, NY: Free Press (2011).

[101] Kirmayer LJ. Psychopharmacology in a globalizing world: the use of antidepressants in Japan. Transcult Psychiatr. (2002) 39:295–322. 10.1177/136346150203900302

[102] Applbaum K. Educating for Global Mental Health. The Adoption of SSRIs in Japan. In: Petryna A Lakoff A Kleinman A editors. Global Pharmaceuticals. Ethics, Markets, Practices. Durham; London: Duke University Press (2006). p 85–110. 10.1215/9780822387916-004

[103] Foucault M. Abnormal: Lectures at the Collège de France, 1974–1975. New York, NY: Picador (2003).

[104] Miller P Rose N. Mobilizing the consumer: assembling the subject of consumption. Theory Cult Soc. (1997) 4:1–36. 10.1177/026327697014001001

[105] Applbaum K. Pharmaceutical marketing and the invention of the medical consumer. PLoS Med. (2006) 3:445–7. 10.1371/journal.pmed.0030189 16597179 PMC1434507

[106] Ignaas D van Hoyweghen I. A new era of medical consumption: medicalisation revisited. Aporia. (2011) 3:16–21. Available online at: http://hdl.handle.net/1854/LU-1852744 (accessed May 19, 2021).

[107] Barsky AJ Borus JF. Somatization and medicalization in the era of managed care. JAMA. (1995) 274:1931–4. 10.1001/jama.1995.03530240041038 8568987

[108] Emanuel EJ Fuchs VR. The perfect storm of overutilization. JAMA. (2008) 299:2789–91. 10.1001/jama.299.23.2789 18560006

[109] Singh I. Doing their jobs: mothering with Ritalin in a culture of mother-blame. Soc Sci Med. (2004) 59:1193–205. 10.1016/j.socscimed.2004.01.011 15210091

[110] Hande MJ Taylor S Zorn E. Operation ASD: philanthrocapitalism, spectrumization, and the role of the parent. In: Burstow B editor. Psychiatry Interrogated. Cham: Springer International Publishing (2016). p. 81–101. 10.1007/978-3-319-41174-3_5

[111] Epstein SG. Patient groups and health movements. In: Hackett EJ Amsterdamska O Lynch M Wajcman J editors. The Handbook of Science and Technology Studies, 3rd Edn, Cambridge, MIT Press (2008). p. 499–539.

[112] Scott WJ. PTSD in DSM-III: a case in the politics of diagnosis and disease. Soc Probl. (1990) 37:294–10. 10.2307/800744

[113] Young A. The Harmony of Illusions: Inventing Post-Traumatic Stress Disorder. Princeton NJ, Princeton University Press (1995). 10.1515/9781400821938

[114] Barker K. Self-Help literature and the making of an illness identity: the case of fibromyalgia syndrome (FMS). Soc Probl. (2002) 49:279–300. 10.1525/sp.2002.49.3.279

[115] Barrett D. Illness movements and the medical classification of pain and fatigue. In: Packard RM Brown PJ Berkelman RL Frumkin H editors. Emerging Illnesses and Society: Negotiating the Public Health. Baltimore, MD, Johns Hopkins University Press (2004). p. 139–70.

[116] Rose SL. Patient advocacy organizations: institutional conflicts of interest, trust, and trustworthiness. J Law Med Ethics. (2013) 41:680–7. 10.1111/jlme.12078 24088159 PMC4107906

[117] Patel V. Where There is no Psychiatrist. A Mental Health Care Manual. Royal College of Psychiatrists, Glasgow: Bell and Bain (2003).

[118] Bauman Z. Globalization. The Human Consequences. Cambridge: Polity Press (1998).

[119] Crouch C. Post-Democracy. Cambridge: Polity Press (2004). 10.1093/0199259402.003.0007

[120] Piketty T. Capital in the Twenty-First Century. Cambridge MA: Harvard University Press (2014). 10.4159/9780674369542

[121] Klein N. This Changes Everything. Capitalism vs. the Climate. New York, NY: Simon & Schuster (2014).

[122] Castner SA Williams GV Goldman Rakic PS. Reversal of antipsychotic-induced working memory deficits by short-term dopamineD1 Receptor stimulation. Science. (2000) 287:2020–2. 10.1126/science.287.5460.2020 10720329

[123] Reilly JL Harris MSH Keshavan MS Sweeney JA. Adverse effects of risperidone on spatial working memory in first-episode schizophrenia. Arch Gen Psychiatr. (2006) 63:1189–97. 10.1001/archpsyc.63.11.1189 17088499

[124] Faber G Smid HGOM van Gool AR Wiersma D Van Den Bosch RJ. The effects of guided discontinuation of antipsychotics on neurocognition in first onset psychosis. Eur Psychiatr. (2012) 27:275–80. 10.1016/j.eurpsy.2011.02.003 21561741

[125] Kirsch I Deacon BJ Huedo-Medina TB Scoboria A Moore TJ Johnson BT. Initial severity and antidepressant benefits: a meta-analysis of data submitted to the Food and Drug Administration. PLoS Med. (2008) 5:e45. 10.1371/journal.pmed.0050045 18303940 PMC2253608

[126] Turner EH Matthews AM Linardatos E Tell RA Rosenthal R. Selective publication of antidepressant trials and its influence on apparent efficacy. N Engl J Med. (2008) 358:252–60. 10.1056/NEJMsa065779 18199864

[127] Read J Cartwright C Gibson K. Adverse emotional and interpersonal effects reported by 1829 New Zealanders while taking antidepressants. Psychiatry Res. (2014) 216:67–73. 10.1016/j.psychres.2014.01.042 24534123

[128] Fava GA Gatti A Belaise C. Withdrawal symptoms after selective serotonin reuptake inhibitor discontinuation: a systematic review. Psychother Psychosom. (2015) 84:72–81. 10.1159/000370338 25721705

[129] Livingston JD Boyd JE. Correlates and consequences of internalized stigma for people living with mental illness: a systematic review and meta-analysis. Soc Sci Med. (2010) 71:2150–61. 10.1016/j.socscimed.2010.09.030 21051128

[130] Chang CR Bassman R. Psychiatric diagnosis and the power of names. J Humanist Psychol. (2019) 59:1–23. 10.1177/0022167819852786

[131] Georgieva I Mulder CL Whittington R. Evaluation of behavioral changes and subjective distress after exposure to coercive inpatient interventions. BMC Psychiatr. (2012) 12:54. 10.1186/1471-244X-12-54 22647058 PMC3412723

[132] Von Peter S. ‘Chronic' identities in mental illness. Anthropol Med. (2013) 20:48–58. 10.1080/13648470.2013.772493 23528064

[133] Haslam N Kvaale EP. Biogenetic explanations of mental disorder: the mixed-blessings model. Curr Dir Psychol Sci. (2015) 24:399–404. 10.1177/0963721415588082

[134] Brinkmann S. Diagnostic Cultures: A Cultural Approach to Pathologization of Modern Life. New York, NY: Routledge (2016). 10.4324/9781315576930

[135] Davies J. Political Pills: Psychopharmaceuticals neoliberalism as mutually supporting. In: Davies J editors. The Sedated Society. Cham: Palgrave Macmillan (2017). p. 189–225. 10.1007/978-3-319-44911-1_8

[136] Hart JT. The Inverse care law, Lancet. (1971) 297:405–12. 10.1016/S0140-6736(71)92410-X 4100731

[137] Wang PS Aguilar-Gaxiola S Alonso J Angermeyer MC Borges G Bromet EJ. Worldwide use of mental health services for anxiety, mood and substance disorders: Results from 17 countries in the WHO World Mental Health (WMH) Surveys. Lancet. (2007) 370:841–50. 10.1016/S0140-6736(07)61414-7 17826169 PMC2847360

[138] Miller D Acton TM Hedge B. The worried well: their identification and management. J R Coll Physicians Lond. (1988) 22:158–65. 3411543 PMC5379321

[139] British Medical Association. Available online at: https://www.bma.org.uk/news/media-centre/press-releases/2018/february/new-bma-research-unveils-blindspot-in-mental-healthcare (accessd at: April 05, 2020).

[140] Davar BV. Globalizing psychiatry and the case of ‘vanishing' alternatives in a neo-colonial state. Disabil Global South. (2014) 1:266–84. Available online at: https://disabilityglobalsouth.files.wordpress.com/2012/06/dgs-01-02-04.pdf (accessed May 19, 2021).

[141] Dorsey ER de Roulet J Thompson JP. Funding of US biomedical research, 2003-2008. JAMA. (2010) 303:137–43. 10.1001/jama.2009.1987 20068207 PMC3118092

[142] Dorsey ER Vitticore P De Roulet J. Financial anatomy of neuroscience research. Ann Neurol. (2006) 60:652–9. 10.1002/ana.21047 17192926

[143] Teddlie C Tashakkori A. Foundations of Mixed Methods Research: Integrating Quantitative and Qualitative Approaches in the Social and Behavioral Sciences. LA: SAGE Publications (2009).

[144] Little TD editor. The Oxford Handbook of Quantitative Methods. Oxford; New York, NY: Oxford University Press (2013). 10.1093/oxfordhb/9780199934898.001.0001

[145] Krueger RA. Focus Groups. A Practical Guide for Applied Research. London: Sage Publications (2015).

[146] Spradley JP. Participant Observation. Wadsworth, Belmont (1980).

[147] Denzin NK Lincoln YS editor. The SAGE Handbook of Qualitative Research, 5th Edn. Thousand Oaks, CA: Sage Publications (2018).

[148] Cargo M Mercer S. The value and challenges of participatory research: strengthening its practice. Annu Rev Public Health. (2008) 29:325–50. 10.1146/annurev.publhealth.29.091307.083824 18173388

